# The Neurochemical Signature of Cardiac Arrest: A Multianalyte Online Microdialysis Study

**DOI:** 10.1021/acschemneuro.4c00777

**Published:** 2025-03-18

**Authors:** C. Cicatiello, S. A. N. Gowers, G. K. Smith, D. Pinggera, S. Orlob, B. Wallner, A. Schiefecker, N. Moser, P. Georgiou, R. Helbok, J. Martini, G. Putzer, M. G. Boutelle

**Affiliations:** † Department of Bioengineering, Imperial College London, London SW7 2AZ, U.K.; ‡ Department of Neurosurgery, Medical University of Innsbruck, Innsbruck 6020, Austria; § Department of Anaesthesiology and Intensive Care Medicine, Medical University Graz, Graz 8010, Austria; ∥ Institute for Emergency Medicine, University Hospital Schleswig-Holstein, Kiel 24105, Germany; ⊥ Department of Anaesthesia and Intensive Care Medicine, Medical University of Innsbruck, Innsbruck 6020, Austria; # Department of Neurology, Medical University of Innsbruck, Innsbruck 6020, Austria; ∇ Department of Electrical and Electronic Engineering and Institute of Biomedical Engineering, Imperial College London, London SW7 2AZ, U.K.; ○ Department of Neurology, Kepler University Hospital, Johannes Kepler University Linz, Linz 4020, Austria; ◆ Clinical Research Institute of Neuroscience, Johannes Kepler University Linz, Kepler University Hospital, Linz 4020, Austria; ¶ Department of Cardiac Anaesthesiology and Intensive Care Medicine, Deutsches Herzzentrum der Charité (DHZC), Berlin 10117, Germany

**Keywords:** online microdialysis, cardiac arrest monitoring, biosensor, ISFET, microfluidics, glucose monitoring

## Abstract

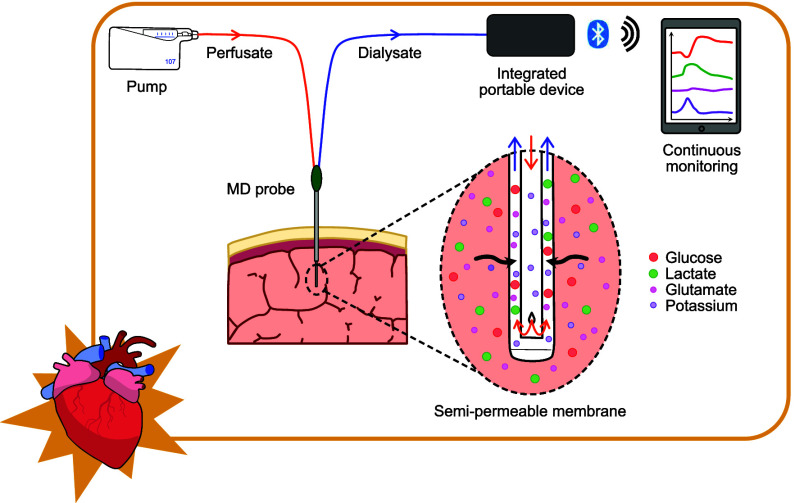

This work describes the use of high resolution online microdialysis coupled with a wireless microfluidic electrochemical sensing platform for continuous monitoring of the effect of cardiac arrest and resuscitation methods on brain glucose and other key neurochemicals in a porcine model. The integrated portable device incorporates low-volume three-dimensional (3D) printed microfluidic flow cells containing enzyme-based biosensors for glucose, lactate and glutamate measurement and a complementary metal-oxide semiconductor (CMOS)-based ion-sensitive field effect transistor (ISFET) for potassium measurement. Both analysis systems incorporate wireless electronics forming a complete compact system that is ideal for use in a crowded clinical environment. Using this integrated system we were able to build a signature of the neurochemical impact of cardiac arrest and resuscitation. Our results demonstrate the almost complete depletion of brain glucose following cardiac arrest and the subsequent increase in lactate, highlighting the vulnerability of the brain while the blood flow is compromised. Following a return of spontaneous circulation, glucose levels increased again and remained higher than baseline levels. These trends were correlated with simultaneous blood measurements to provide further explanation of the metabolic changes occurring in the brain. In addition, the onset of cardiac arrest corresponded to a transient increase in potassium. In most cases glutamate levels remained unchanged after cardiac arrest, while in some cases a transient increase was detected. We were also able to validate the trends seen using online microdialysis with traditional discontinuous methods; the two methods showed good agreement although online microdialysis was able to capture dynamic changes that were not seen in the discontinuous data.

## Introduction

Cardiac arrest remains one of the leading causes of death and disability worldwide, placing a significant burden on healthcare systems and on the economy. According to the American Heart Association (AHA), sudden cardiac arrest is responsible for approximately 436,000 fatalities per year in the United States.^1^ This number exceeds the projected deaths from the five highest mortality cancers combined (lung, colorectal, pancreatic, breast and prostate) in 2024.^2^ Every year in the United States there are 300,000 cases of in-hospital cardiac arrest, with a survival rate of just 25%,^3^ as well as 365,000 cases of out-of-hospital cardiac arrest, with a survival rate of approximately 10%.^4^

The abrupt loss of cardiorespiratory function during cardiac arrest leads to a cessation of cerebral blood flow, resulting in cerebral hypoxia and a rapid loss of consciousness. Restoring spontaneous circulation as soon as possible is vital to ensure long-term survival and good functional outcome.^3^ The provision of effective and early resuscitation strategies such as cardiopulmonary resuscitation (CPR) can improve the prognosis of cardiac arrest patients by two to four times.^5^ CPR is a lifesaving intervention consisting of chest compressions aimed at restoring the blood flow after cardiac arrest; it is described by the AHA as the most critical procedure in emergency cardiovascular care.^6^ Standard resuscitation methods also include electrical defibrillation of the heart and adrenaline administration in an effort to obtain a return of spontaneous circulation (ROSC). Despite progress in resuscitation research, high-quality CPR remains inefficient, providing only 30–40% of physiological cerebral blood flow,^1^ which is below the 40–50% threshold required to prevent neuronal cellular injury.^7^

The brain receives 15–20% of the total cardiac output to maintain tissue perfusion and metabolic homeostasis.^7^ As a result, neuronal tissue viability relies heavily on glucose supply and is therefore at extremely high risk during cardiac arrest. Reports indicate that 70% of patients admitted to hospital following an out-of-hospital cardiac arrest die from hypoxic–ischemic brain injury even when initial CPR is successful, and 19% of those who survive until discharge are left with mild to severe neurological impairments.^5^ Consequently, post cardiac arrest care focuses primarily on minimizing neurological injury.^8^ Nevertheless, a knowledge gap remains regarding the development of brain injury during cardiac arrest and resuscitation care.^8^ In light of this, combining cardiac output monitoring with neuromonitoring can be an effective strategy in elucidating the pathological cascade associated with primary and secondary brain damage. In particular, neurochemical monitoring is a valuable tool that can be used to measure molecular changes in brain tissue,^9−11^ and to assess the efficacy of treatments in preclinical stages.

In this context, microdialysis (MD) is a powerful tool as it is an FDA-approved sampling technique with a wide application in neurocritical care settings. MD enables probing of the tissue neurochemistry and the simultaneous analysis of multiple analytes of interest. The MD probe is a minimally invasive catheter that is continuously perfused with a solution mimicking the composition of the extracellular fluid. The probe consists of two concentric tubes connected at the distal end by a semipermeable membrane, which allows for diffusion of molecules from the extracellular fluid into the probe. The concentration of an analyte in the dialysate sample is representative of that in the interstitial fluid. MD has proved to be very helpful in the context of brain injury studies, particularly in the case of traumatic brain injury (TBI)^12^ and subarachnoid hemorrhage (SAH).^13^ Neurochemical monitoring with MD has highlighted the critical balance between cerebral energy metabolism and cerebral blood flow^14^ with studies demonstrating that both low and high dialysate glucose levels are associated with unfavorable outcomes.^15^ Hence, it is of interest to broaden the scope of MD to investigate the effect of impaired blood supply during cardiac arrest and resuscitation on cerebral metabolism.

Previous studies have used MD to investigate the effect of cardiac arrest and resuscitation on the brain and showed metabolic changes indicative of ischemia.^16−19^ However, these studies used traditional discontinuous MD sampling resulting in low time resolution, typically from every 8 min to hourly. Traditional MD involves the collection of dialysate samples into vials for offline analysis using benchtop instruments. As samples are discrete, the time resolution is limited by how often the vials are replaced. However, MD can be coupled with continuous analysis systems to enable the continuous detection of analytes in real time with improved time resolution. Online MD can therefore be used to characterize complex fast dynamic processes such as neuronal depolarization events.^20^ The major challenge in transitioning from discontinuous to online MD lies in the miniaturization of the analysis device, which needs to be placed close to the patient in order to minimize delays due to long connection tubing and be small enough not to interfere with clinical procedures. Advancements in the field of microfluidics, biosensors and electronics have made the development of miniaturized and portable biosensing systems possible for the continuous and real-time analysis of MD streams.^21,22^ We have previously reported the use of online MD to describe the dynamics of cerebral pathophysiology and the onset of spreading depolarizations (SDs) in TBI patients in intensive care units.^20^ In addition we have demonstrated the feasibility of using online MD to detect real-time metabolic changes in the brain during cardiac arrest in a proof-of-concept study.^23^

Here we report the use of a wireless microfluidic electrochemical sensing platform to measure the effect of cardiac arrest and resuscitation on brain glucose and other key analytes, including lactate, glutamate and potassium, in real time in a multimodal neuromonitoring porcine model. The gyrencephalic anatomy of the porcine brain makes it an ideal model for the human brain, facilitating the translation of these study results into clinically relevant outcomes. In this study the newly optimized monitoring device presented is coupled with online MD to continuously sample the brain interstitial fluid. Changes in brain glucose and other analytes in the resulting dialysate are monitored with high time resolution to help elucidate the neurochemical changes occurring during cardiac arrest and resuscitation.

## Results and Discussion

We have previously developed analysis systems for the individual monitoring of metabolites^23^ and ions.^24^ In this study we combine them to create a new integrated portable system, with the addition of glutamate, for continuous online dialysate monitoring. The portable analysis system was necessary in this case to provide a flexible setup to allow us to work around the continuous clinical preparation taking place. During the experimental setup, complex surgical procedures were carried out requiring many people. It was a huge advantage that we were able to calibrate the analysis system out of the way and monitor it remotely and later move the system to be by the animal’s head without having to disconnect anything.

**Figure 1:**
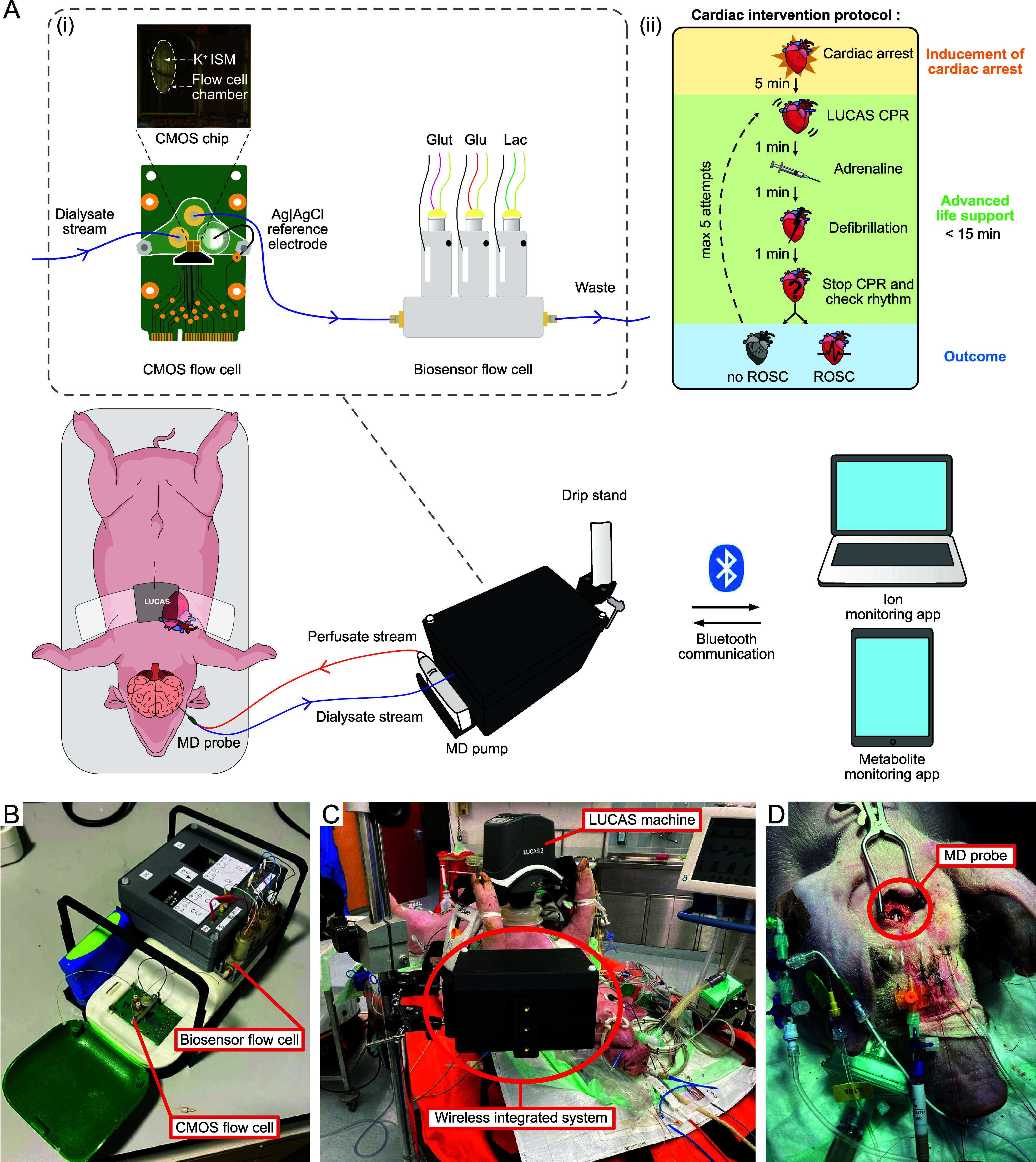
Experimental setup (A) Illustrative schematic of the experimental setup in the case of the one probe configuration. The anesthetized pig was placed on the operating table. The MD probe was inserted into the brain, and perfused with sterile perfusion fluid at 1 μL/min. The MD probe was inserted on average 3 h before baseline measurement commenced. (i) The outlet of the MD probe was connected to our wireless integrated neurochemical system, which consisted of a CMOS chip coated with a potassium ion selective membrane (ISM) and of a biosensor flow cell housing a glutamate, a glucose and a lactate biosensor. The dialysate concentration was monitored continuously and in real time throughout the experiment. The potentiometric CMOS chip (potassium) and amperometric biosensors (glucose, lactate and glutamate) communicated via Bluetooth with custom-made apps running on a portable laptop and tablets, respectively. (ii) Baseline levels were monitored for at least 30 min prior to commencing the experimental protocol. The experimental protocol began by inducing cardiac arrest followed by a 5 min “no-touch” period. The advanced life support protocol was then followed consisting of CPR using a LUCAS device (LUCAS3, Stryker, Portage, MI) and iterative resuscitation steps of adrenaline injections and defibrillations for a period of up to 15 min until the pig achieved ROSC. If ROSC was not achieved after 15 min, the resuscitation steps were terminated. In the case of ROSC, the monitoring continued for a further 2 h, at the end of which time the pig was euthanized. (B) Photo of the wireless integrated neurochemical system with the CMOS flow cell and the biosensor flow cell. (C) Photo of the pig positioned on the operating table underneath the LUCAS machine with the wireless integrated system box positioned on a drip stand close to the bedside. (D) Photo of the surgical setup. MD probe and catheter placement was performed via a burr hole. Full experimental details are given in the methods section at the end of the manuscript.

The system could take dialysate input from one or two microdialysis probes. Figure 1A shows the experimental setup for the one probe configuration. A 3D printed box was designed, shown in Figure 1B,C, to hold the combined amperometric biosensors (for glucose, lactate and glutamate measurement) and potentiometric CMOS chip (for potassium measurement) securely in place. It also included a slot to hold the MD pump, which would otherwise be loose on the operating table and at risk of damage. The box was secured to a drip stand, which allowed the box to be placed close to the pig’s head without obstructing the overall experiment. It also made it easy to move the box during the experiment as needed.

Both amperometric and potentiometric platforms allowed the data to be transmitted wirelessly; the three channels of biosensor data were displayed on tablets using a custom-made Android app, and the CMOS data was displayed on a laptop using a custom-made Matlab app. This allowed continuous visualization of the data, which was vital to check the operation of the sensors and track changes in the analyte concentrations, away from the otherwise crowded operating table. The integrated portable system was successfully taken abroad and proved to be robust and capable of simultaneously monitoring multiple analytes for the first time.

**Figure 2:**
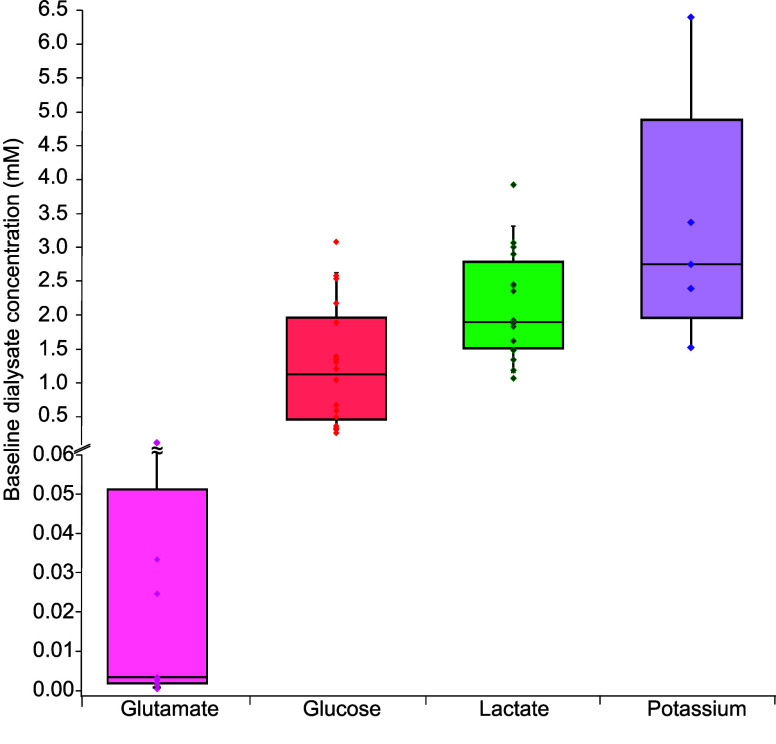
Baseline box plots. Box plots showing baseline dialysate levels of glutamate (pink, *n* = 10), glucose (red, *n* = 18), lactate (green, *n* = 16) and potassium (purple, *n* = 5) at 1 μL/min, measured as the mean of the signal in the 5 min window immediately before the start of the cardiac arrest protocol. Box plots show the median and interquartile range and whiskers represent the 10th and 90th percentiles. For glutamate, the upper whisker is truncated to zoom in on the box plot as one value was much higher than the other values. Actual points overlaid on top of box plots.

Using this integrated system we reliably monitored glucose levels in 18 individual pigs. From these cases we could determine the median baseline levels of each analyte in the brain dialysate before any intervention took place. Box plots representing the baseline dialysate levels for each analyte are shown in Figure 2. We found the median baseline level to be 1.13 (0.46–1.96) mM for glucose (*n* = 18) and 1.90 (1.52–2.79) mM for lactate (*n* = 16). We also found the median baseline level to be 3.41 (1.86–51.3) μM for glutamate (*n* = 10) and 2.75 (1.96–4.89) mM for potassium (*n* = 5) (median and interquartile range). To carry out the simultaneous online measurement of multiple analytes, the integrated analysis system had to be optimized in parallel with the animal experiments. As a result, there are a lower number of measurements for glutamate and potassium. It is noted that there is a high variability in the glutamate levels. The variability is not due to trauma caused by insertion of the MD probe as these measurements were made several hours after probe insertion. However, this may reflect the sensitivity of the glutamate system to anesthesia and other clinical procedures.^25,26^

It is interesting to compare these baseline levels with those previously seen. To make these comparisons, any differences in MD flow rates need to be considered as a lower flow rate will result in a higher probe recovery and hence a higher concentration of analyte in the dialysate. Taking this into account, the baseline levels of glucose and lactate seen in this study at 1 μL/min are comparable with those previously seen by Putzer et al. in another porcine study following a very similar clinical protocol at a higher flow rate of 2 μL/min.^18^

Averaging measurements together over a specific time window is very useful for comparing specific time points across multiple cases, as shown in Figure 2 for the baseline measurements. However, a unique aspect of real-time data monitoring is that the traces are much more individualized, both in terms of the magnitudes of change and, in particular, their relative timing. While this variability can be minimized through strict experimental protocols, these inherent differences cannot be completely eliminated. The ability to measure continuous data provides a level of detail that would be lost if the data were averaged across all pigs.

**Figure 3:**
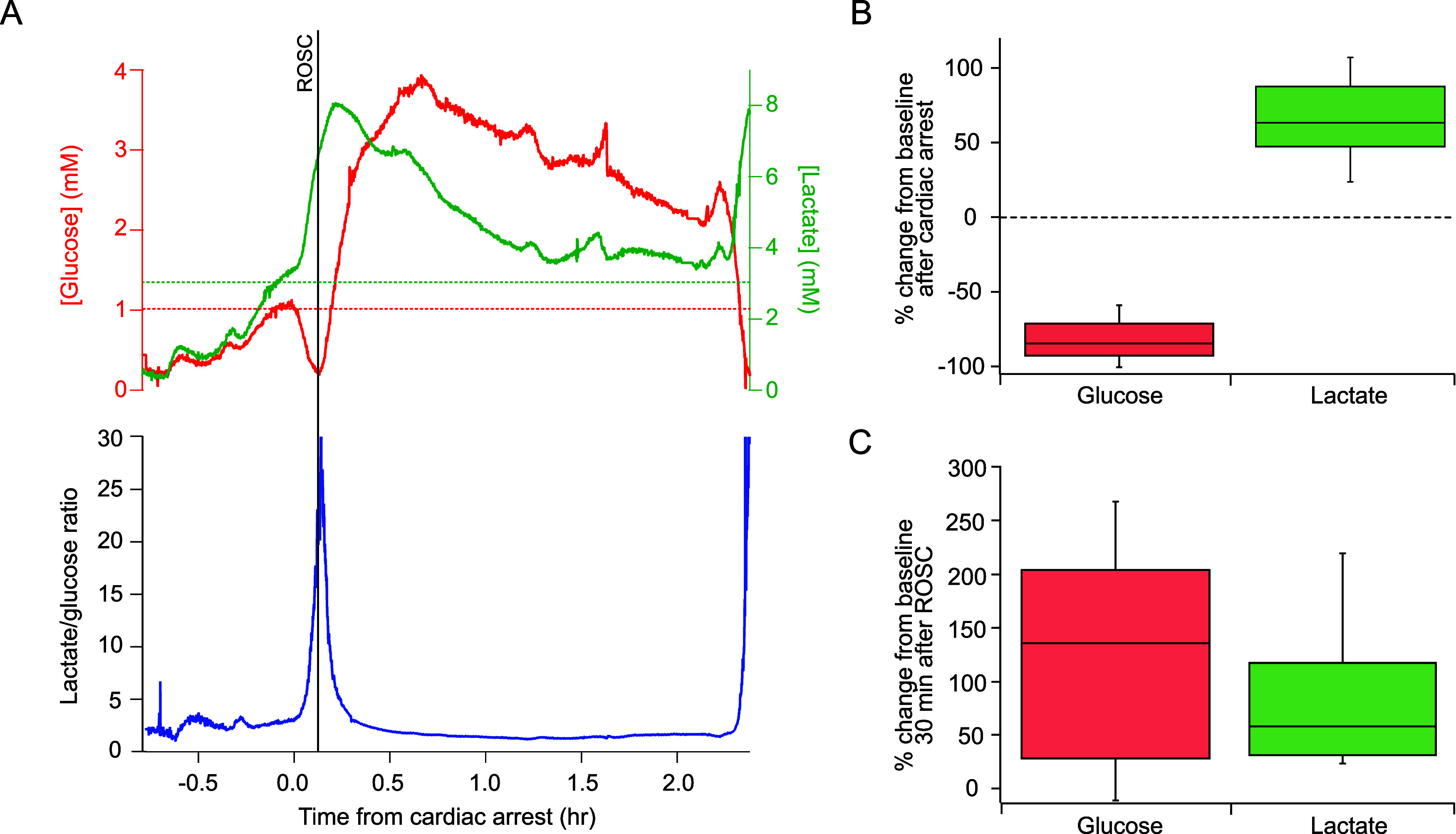
Brain dialysate glucose and lactate traces during the cardiac arrest intervention protocol. (A) Example of the brain dialysate glucose level (red), lactate level (green) and the lactate/glucose ratio (blue) during the protocol. The data were sampled at 10 Hz, smoothed with a Savitzky–Golay 201-point filter and shifted to align time 0 with the time of cardiac arrest. The vertical line indicates the time of ROSC. At the end of the protocol the pig was euthanized with an infusion of potassium chloride (KCl). The horizontal dotted lines indicate the levels taken as baseline for glucose (red) and lactate (green) in this case. This was calculated as the mean of the signal in the 5 min window immediately before the start of the cardiac arrest protocol. (B) Box plots showing the percentage change in glucose (red, *n* = 16) and lactate (green, *n* = 14) from baseline after cardiac arrest. Glucose traces always showed a clearly defined trough following cardiac arrest, so levels were taken when they were at a minimum. For lactate, as the increase was less clearly defined between pigs, we chose to evaluate the level at 6 min after cardiac arrest to enable comparison. The 0% (horizontal dotted line) represents baseline levels before cardiac arrest, while -100% represents complete depletion of the brain glucose. (C) Box plots showing the percentage change 30 min after ROSC compared with baseline for glucose (red, *n* = 8) and lactate (green, *n* = 7) for cases where ROSC was achieved. In all cases, boxes represent median and interquartile range and whiskers represent the 10th and 90th percentiles.

The high temporal resolution of online MD allows us to identify the typical response in neuronal glucose and lactate to the different phases of the cardiac intervention protocol: cardiac arrest, CPR, ROSC, and death. Figure 3A shows an example of the brain dialysate glucose and lactate levels during the experiment and the striking effect cardiac arrest has on cerebral metabolism. Since glucose and lactate change in opposite directions during ischemic events the lactate/glucose ratio has been shown to be a sensitive and reliable indicator of tissue health as it is not affected by artifacts caused by changes in MD probe recovery;^27^ this is also shown in Figure 3A. In this case there is relatively large drift in the baseline values before cardiac arrest; in the 30 min before cardiac arrest glucose changed on average by 0.24 ± 0.22 mM and lactate changed by 0.42 ± 0.64 mM across all the cases monitored, whereas here glucose changed by 0.75 mM and lactate by 2.46 mM. As the MD probe was implanted at least 3 h before monitoring began this is unlikely to be due to tissue stabilization effects following implantation, which we have previously found to be less than 15 min,^28^ and instead seems to reflect real changes occurring in the brain.

At the onset of cardiac arrest the level of glucose dramatically decreased and the level of lactate increased. The lactate/glucose ratio also increased sharply at cardiac arrest. If ROSC occurred (as is the case for the example shown in Figure 3A), the trends for glucose and lactate were reversed; in the 2 h period after ROSC the level of lactate slowly decreased though did not completely return to the baseline level. In contrast, the level of glucose increased and remained at a value higher than baseline. This high level of glucose was sustained throughout the post-ROSC period.

A summary of the changes in glucose and lactate seen at cardiac arrest across all the cases monitored is shown in the box plot in Figure 3B. The percentage change when glucose was at a minimum following cardiac arrest compared with baseline confirms the profound effect of cardiac arrest on the brain tissue. The median percentage change in glucose was −84.4% (IQR = −92.8 to −71.3%) (*n* = 16), where −100% indicates complete depletion. The percentage change from baseline in lactate measured 6 min after cardiac arrest is characterized by a higher degree of variability between pigs compared with that of glucose. Nevertheless, in all cases the lactate increased after cardiac arrest and the median percentage change was of 63.8% (IQR = 48.1–88.1%) (*n* = 14). These changes illustrate the immediate and dramatic effect of cardiac arrest on the brain, resulting in the almost complete depletion of tissue glucose. This coupled with the lack of oxygen delivery, ultimately causes the metabolism to be dominated by anaerobic respiration leading to an increase in tissue lactate. Our results suggest that due to its high metabolic activity, the brain is particularly vulnerable to global ischemic events. Figure SI1 confirms the hypoxic conditions of brain tissue following cardiac arrest, showing a typical trend of brain tissue oxygen partial pressure (PbtO_2_) during the cardiac intervention protocol.

The effect of ROSC on the brain metabolism across all the cases monitored is summarized in the box plot in Figure 3C, which shows the percentage change in the glucose and lactate levels recorded 30 min after ROSC compared with baseline levels. The median percentage change in glucose after ROSC was 135.6% (IQR = 28.0–203.9%) (*n* = 8). These results show that the percentage change in glucose after ROSC was quite variable, however, in all but one case, the glucose levels were higher 30 min after ROSC compared with baseline. The median percentage change in lactate after ROSC was 57.9% (IQR = 31.0–116.8%) (*n* = 7), demonstrating that lactate levels were also elevated 30 min after ROSC compared with baseline, but to a lesser extent than for glucose. This increased glucose following ROSC could be explained by increased supply due to temporary hyperaemia, with lactate levels also elevated due to increased glucose availability. Alternatively, the glucose increase could be explained by a release of stress hormones leading to high serum glucose levels or could be attributed to the effect of adrenaline on the heart. Glucose levels only increase when ROSC is achieved, demonstrating that CPR alone does not provide sufficient blood flow to reverse the effects of cardiac arrest on the brain.

At the end of the monitoring period the pig was euthanized with an intravenous (IV) injection of potassium chloride. As seen in Figure 3A at around 2.25 h after cardiac arrest, the dialysate glucose level dropped to zero and the level of dialysate lactate increased due to continued glucose consumption and anaerobic glycolysis after cardiac arrest.

To evaluate the impact of ischemia on the brain, it is interesting to compare the rate of decrease in glucose measured in this study in the brain with that seen in both the human brain and in peripheral tissue in other studies conducted using similar monitoring systems. As each study used different MD flow rates and probes, and each tissue had different resting glucose levels, we have calculated the percentage change per minute in dialysate glucose taking resting glucose as baseline. Table 1 gives examples of the percentage change in dialysate glucose per minute in an example case from three different applications where peripheral tissue was monitored after the blood supply was terminated; these applications included monitoring in the human leg during an operation where the femoral artery was temporarily clamped (unpublished work), monitoring in free flap tissue as it was moved from the donor to the recipient site,^29^ and monitoring in a porcine leg as cardiac arrest was induced.^23^ These rates of change are compared with the rate of glucose decrease seen in the brain after cardiac arrest and at death for one pig in this study. As seen in Table 1 in all cases there is a decrease in glucose at the onset of ischemia. In the peripheral tissue this decrease in glucose is relatively slow, in the range of 1.5–2.6%/min. However, in the brain the rate of glucose decrease is markedly faster; in this example a 17.7%/min decrease was seen at cardiac arrest and a 43.6%/min decrease was seen when IV potassium chloride was given, which would have stopped the heart and additionally caused brain depolarization. We also include for completeness an example of the rate of glucose decrease in a TBI patient during a secondary brain insult (spreading depolarization) where the rate of decrease was 10.3%/min.^20^

**Table 1:** Percentage Decrease in Dialysate Glucose Per Minute for an Example Case in Different Monitoring Applications^a^

monitoring application	percentage decrease in glucose per min
human leg after femoral artery clamp	2.6 ± 2.0%
human free flap^29^	2.6 ± 0.1%
porcine leg after cardiac arrest^23^	1.5 ± 0.3%
human secondary brain insult^20^	10.3 ± 0.6%
porcine brain after cardiac arrest	17.7 ± 0.2%
porcine brain after IV potassium chloride	43.6 ± 1.0%

a The error is calculated by propagating the standard deviation of the levels before and after the change.

This comparison provides clear evidence that the brain is much more vulnerable to changes in glucose availability compared with peripheral tissue and highlights the dramatic impact of even relatively short ischemia on the brain.

**Figure 4:**
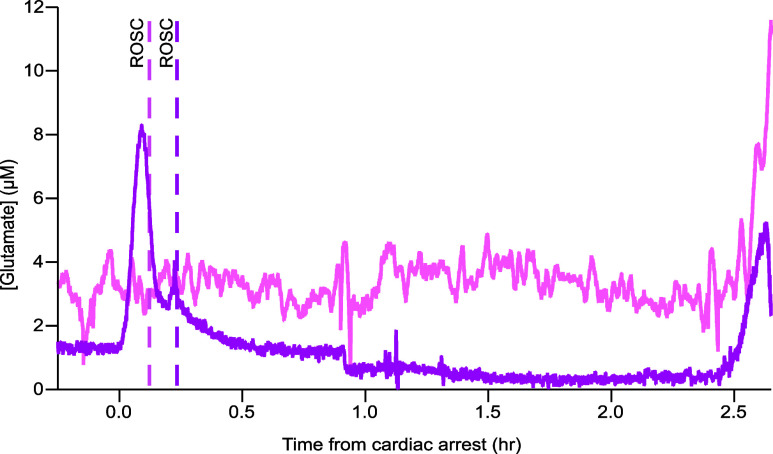
Brain dialysate glutamate response during the cardiac intervention protocol. Two examples of the brain dialysate glutamate response during the cardiac intervention protocol representing the two trends observed. The data were shifted to align time 0 with the time of cardiac arrest. Each vertical line indicates the ROSC time for the case associated with the same color. In one case (magenta) there was a transient peak in glutamate in response to cardiac arrest, whereas in the other (light pink) the glutamate level remained unchanged. The increase at 2.5 h after cardiac arrest in both traces corresponds to death. The two sets of data were sampled at 10 Hz, smoothed with a Savitzky–Golay filter (201-point for magenta and 1501-point for light pink).

In addition to glucose and lactate, we also monitored glutamate in the brain dialysate. In general, glutamate levels remained stable throughout cardiac arrest and resuscitation, although in two of the cases monitored a sharp increase was seen at the onset of cardiac arrest, which returned to baseline before ROSC was achieved. Figure 4 shows an example of the typical dialysate glutamate levels seen during the cardiac protocol for the majority of the cases (light pink) as well as one of the cases where a transient increase is seen at cardiac arrest (magenta). This result was surprising as we would have expected a consistent increase in glutamate at cardiac arrest and other studies have shown such an increase.^19^ However, regulation of extracellular glutamate is highly complex and strongly related to the energy state of the tissue. A clear explanation of the two trends observed is therefore not trivial and we are investigating this further.

In 80% of the cases monitored, the glutamate level increased when the pig was euthanized, as shown in both examples in Figure 4. This can be explained by the fact that the lethal administration of potassium chloride reduces the electrochemical gradient for potassium in the heart muscle, leading to immediate cardiac arrest, and causes rapid disruption of the neuronal activity resulting in increased glutamate levels.

**Figure 5:**
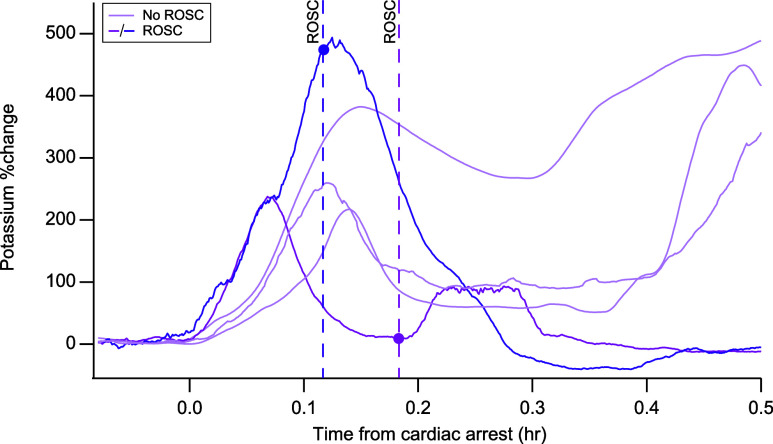
Overlay of the normalized brain dialysate potassium traces following cardiac arrest. The percentage change in potassium from baseline is shown for five different cases. The data were shifted to align time 0 with the time of cardiac arrest. Each vertical line indicates the ROSC time for the case of the same color. ROSC was not achieved in 3 cases (light purple). The data were sampled at 0.3 Hz and were filtered using a Savitzky–Golay filter (25-point).

Dialysate potassium levels were also measured in five cases, as shown in Figure 5 as a percentage change from baseline. In all five cases a transient increase in potassium was observed at the onset of cardiac arrest, indicative of brain depolarization. ROSC was achieved in two of the five cases where potassium was monitored. Interestingly, for the two cases where ROSC was achieved, the level of potassium decreased back to near baseline levels following ROSC, indicated by the correspondingly colored dotted lines in Figure 5. In contrast, for the cases where ROSC was not achieved, after the transient increase the level of potassium did not return to baseline and later increased further when CPR stopped. This initial assessment suggests that measuring potassium could be useful in identifying brain ischemia. We are currently carrying out further experiments to investigate this further.

In addition to the online MD probe already described, another MD probe was also inserted into the brain allowing us to validate our online analysis system against traditional discontinuous MD (MDialysis, Stockholm, Sweden). While the outlet of the online MD probe was connected to our analysis system for continuous measurement, discrete dialysate samples were collected in sample vials from the outlet of the offline MD probe. These discontinuous samples were frozen at −20 °C and analyzed within two to 4 weeks using an ISCUSflex microdialysis analyzer (MDialysis, Stockholm, Sweden). To achieve a good compromise between high probe recovery and reduced time delay in the dialysate reaching our online analysis system, the online MD probe was perfused at 1 μL/min. However, for the discontinuous method, the MD probe was perfused at a higher flow rate of 2 μL/min to ensure the collection of sufficient analysis volume while achieving a reasonably good time resolution. These differences in perfusion flow rates will result in differences in MD probe recovery, however, by expressing the measurements as a percentage change from baseline, probe recovery differences are removed and the two methods can be compared.

**Figure 6:**
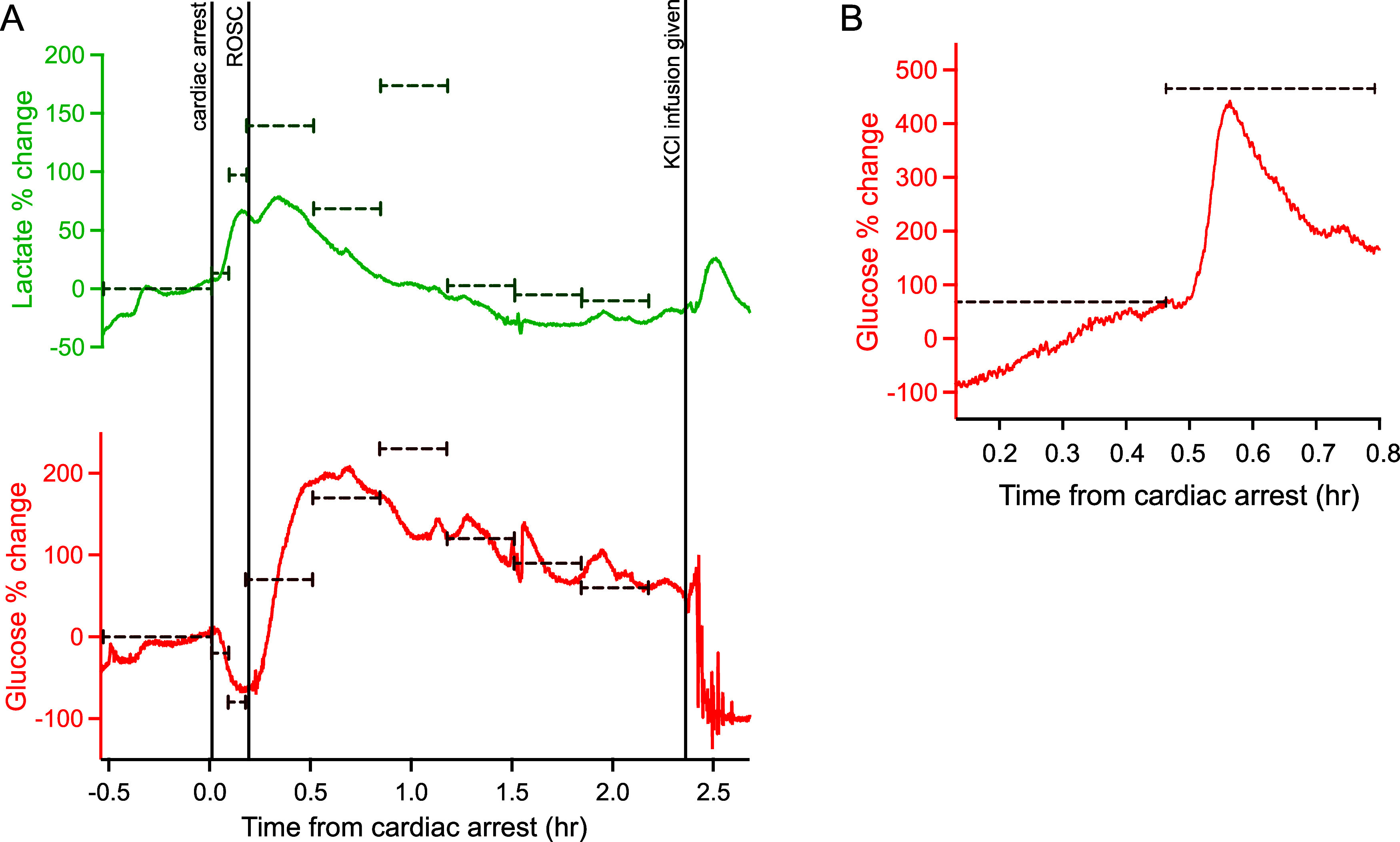
Online microdialysis measurements compared with discontinuous measurements. (A) Dialysate percentage change from baseline for glucose (red) and lactate (green) measured using the continuous online analysis system compared with offline analysis of discontinuous samples. Vertical lines indicate key time points in the protocol. (B) Another example of percentage change from baseline for glucose measured using the continuous online analysis system compared with discontinuous offline analysis for a second pig. In all cases the online MD probe was perfused at 1 μL/min and the offline probe was perfused at 2 μL/min. Any difference in probe recovery due to the different flow rates for the two methods is removed in the percentage change. Discontinuous offline measurements are represented by horizontal dotted lines, indicating the time that measurement was collected over.

Figure 6A shows exemplar data from an individual case comparing online and offline glucose and lactate percentage changes over the course of a typical experiment. The continuous traces show the measurements obtained for glucose (red) and lactate (green) using our online analysis system. The dashed horizontal bars show the corresponding offline measurements. The bar lengths indicate the time over which the sample was collected. As shown in Figure 6A, the continuous online measurements follow those recorded using offline MD closely for both glucose and lactate in terms of trend and percentage change from baseline. A discrepancy between the offline and online levels recorded for both glucose and lactate is seen at around 0.75 h from cardiac arrest, which could be due to an anomaly in the offline sample as the samples either side agree closely with the online results. Figure 6B compares an excerpt of online and offline glucose data for another experimental case. As shown here, although the overall trends are consistent between the two methods, the online measurement is able to resolve a dynamic event that is averaged out in the offline data. We conclude from this that general trends are closely aligned between the two methods, but online MD offers much greater resolution in detecting transient changes.

**Figure 7:**
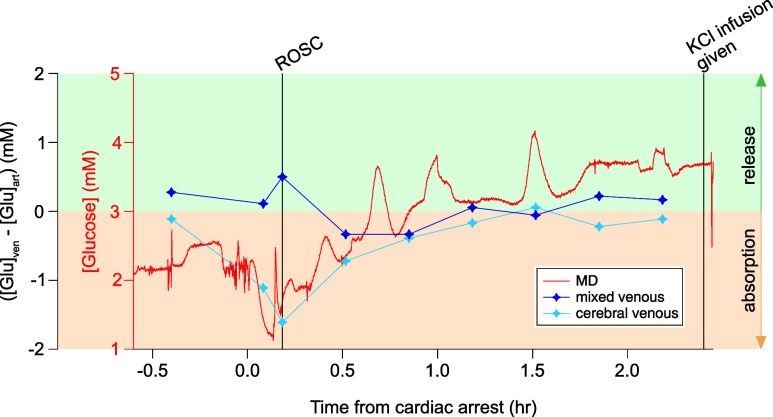
Comparison of glucose levels in the dialysate with the net change in glucose between arterial blood samples and venous blood samples. The continuous MD data for glucose (in red) is compared with the changes in glucose in the blood samples that were taken at regular intervals during the clinical procedure and measured using a blood gas analyzer: at baseline, at the start of CPR, at ROSC, and afterward every 20 min until the end of the procedure. In more detail, the markers represent the difference between the glucose in the arterial blood sample and the venous blood sample, either mixed venous or cerebral venous, for each time frame. A positive value refers to a release of glucose from the tissue into the blood, while a negative value refers to an absorption of glucose into the extracellular space from the blood.

To try and understand the metabolic changes seen in the dialysate it is interesting to look at the difference between the levels of glucose in the arterial blood going into the brain and in the venous samples coming out of the brain throughout the experiment. Figure 7 shows the brain dialysate glucose level for one case compared with the net blood glucose in the cerebral-venous blood samples and in the mixed-venous blood samples, obtained by subtracting the arterial glucose concentration from the venous glucose concentration (either cerebral or mixed venous): a positive value corresponds to a release in glucose from the tissue to the bloodstream and a negative value corresponds to the absorption of glucose into the extracellular space from the bloodstream. The original arterial and venous measurements are shown in the Supporting Information, Figure SI2.

The trend in cerebral blood glucose levels mirrors the general trend seen in the dialysate data. There was a net decrease in the cerebral blood glucose level after the cardiac arrest (second and third samples, light blue markers) followed by a net increase after ROSC (fourth sample). This is due to increased delivery of glucose combined with reasonable consumption as shown in Figure SI2; this is indicative of healthy well-regulated tissue and therefore successful ROSC, suggesting that this is a good experimental model for cardiac arrest. Around 2 h after ROSC, there is no net change between the arterial and cerebral-venous blood glucose measurements. In this example, transient increases are seen in the dialysate glucose data after ROSC, which are not seen in the blood measurements due to the lower temporal resolution of the discontinuous blood measurements. We believe such transient increases in tissue glucose would be consistent with induced transient hyperemic events such as spreading depolarizations in otherwise healthy tissue with normal blood supply. This requires further investigation before any conclusions can be drawn.

The net glucose concentration in the mixed-venous blood measurements was relatively stable throughout the clinical intervention, confirming that other organs and peripheral tissues are much less susceptible to metabolism impairments compared with the brain. The net increase in glucose in the peripheral blood measurement at ROSC (third sample) is caused by a higher level of glucose in the mixed-venous blood sample compared with the arterial blood sample. This could be explained by the fact that the peripheral tissue has sufficient stores of carbohydrate that can be used to produce glucose in response to ischemia.^30^ The ability to correlate the glucose trend seen in the extracellular fluid with the net change between the arterial and venous blood samples allows us to begin to build a picture of what is happening in the brain during the experimental protocol.

Potassium was also measured in the blood, however, the correlation between systemic and MD data for potassium is more challenging as potassium transport across the blood brain barrier is mediated by ion transporters. Figure SI3 shows exemplar blood potassium levels for one pig during the experimental protocol. It is particularly interesting to note that the potassium increase at cardiac arrest is much larger in the brain than in the blood, indicating that potassium levels must be increasing in the brain tissue itself presumably due to mass depolarization, as the increase cannot be explained by the increased levels in the blood alone.

## Conclusions

In this study, we have demonstrated the use of a combined multianalyte clinical system to monitor neurochemical changes in a porcine model of cardiac arrest and resuscitation. The portable system was transported abroad and easily assembled on site where it was successfully used to reliably monitor glucose and other key analytes in a pseudoclinical environment. The small size of the combined system and the wireless connection were significant advantages, making this device well-suited for a crowded operating theater with numerous instruments and a large medical team working on the cardiac intervention protocol. Importantly, the small size also meant that the monitoring system could be placed close to the pig reducing the need for long connection tubing and therefore minimizing the delay between the MD probe and the analysis system.

This study demonstrates the first-ever simultaneous online monitoring of neuronal glucose, lactate, glutamate and potassium in real time during cardiac arrest and the early postresuscitation period in an animal model. Using this system we were able to resolve dynamic neurochemical changes occurring during cardiac arrest, reperfusion and death with high time resolution. Our results demonstrate the almost total depletion of glucose occurring in the brain following cardiac arrest and even during resuscitation efforts. This highlights the severe effect that cardiac arrest can have on the brain and the inefficiency of current CPR protocols in delivering sufficient blood supply to the brain to meet its energy demands. We found that after ROSC glucose levels increased and remained elevated compared with baseline levels.

We also reported the rapid occurrence of a transient potassium increase after cardiac arrest, indicative of neuronal depolarization, with levels returning to baseline only if ROSC occurred. These preliminary results suggest that potassium could be used as an indicator of brain ischemia. Future work will focus on investigating this further. In the majority of cases we found that glutamate levels remained stable during cardiac arrest and CPR and only increased at the time of death. Additional research on glutamate changes during cardiac arrest is ongoing to better understand the effect of ischemia on neuronal activity and neurotoxicity.

The measurements obtained with our novel online MD system were validated against those obtained with a traditional discontinuous MD approach, where the dialysate samples were analyzed offline with a gold-standard commercially available instrument. We found there was good agreement between the trends in the online and offline measurements. However, the online system was able to detect rapid transient changes with high time resolution, which were not resolved with the conventional offline approach. This demonstrates the greater suitability of online MD systems in characterizing the dynamics and evolution of the complex pathological phenomena underlying the development of brain damage following cardiac arrest.

In order to better understand the trends in glucose seen in the extracellular fluid, we were able to compare the dialysate measurements with blood measurements. There was a similar trend in the net difference between arterial and cerebral-venous samples and the brain dialysate glucose, demonstrating that measuring tissue glucose using MD can be used to probe the balance between glucose availability and usage.

The results of this proof-of-concept study demonstrate the potential of this novel multianalyte clinical system to understand the neurochemical changes occurring during cardiac arrest and resuscitation and hence guide the advancement of emergency cardiovascular care. We are currently conducting experiments to investigate neurochemical changes during prolonged resuscitation efforts. Future improvements would include addition of a null sensor to further minimize the effect of interferents especially for glutamate which is present at μM levels. Further integration of all sensors into a single microfluidic device would improve issues with back pressure (due to connectors) and reduce the delay between sensors. Further miniaturization of the analysis system would also allow it to be placed even closer to the bed, which would reduce the probe delay. The purpose of this study was not to develop a tool for use in humans during cardiac arrest but rather to understand the chemical changes occurring in the brain after cardiac arrest and to evaluate novel resuscitation methods. However, in the future this methodology could provide a useful monitoring tool for comatose patients who survive the acute phase of cardiac arrest, facilitating the assessment of brain injury and the prediction of neurological outcomes.

## Methods

### Experimental Section

#### Reagents

Glucose oxidase (GOx) from*Aspergillus niger*and lactate oxidase (LOx) from*Aerococcus viridans*were purchased from Sekisui Diagnostics. l-Glutamate oxidase from recombinant*Escherichia coli*was purchased from Cosmo Bio Co. All other reagents were obtained from Sigma-Aldrich.

#### Electrode Fabrication

The biosensors used in this study were fabricated based on a combined needle 3-electrode system described elsewhere.^31,32^ Briefly, they were made by threading a 50 μm diameter poly(tetrafluoroethylene)-insulated platinum/iridium wire (90:10) (Advent Research Materials Ltd., U.K.) and a 50 μm polyester-insulated silver wire (Goodfellow Cambridge Ltd., U.K.) through a 27G needle. Electrical wires were attached using conductive silver epoxy (RS Components Ltd., U.K.). The needle barrel and shaft were filled with epoxy resin (CY1301 and HY1300, Robnor ResinLab Ltd., U.K.) to support the wires. The needle tip was made blunt using sandpaper (Buehler Ltd., USA) and further polished using 1, 0.3, and 0.05 μm alumina slurries to create two 50 μm diameter disc electrodes at the tip. The platinum disc was used as the working electrode. The silver disc was chloridized by placing the electrode in a 1 M potassium chloride solution and applying +0.45 V to the silver wire vs a standard Ag|AgCl reference electrode (BASi, USA) for 15 min to yield an Ag|AgCl pseudo reference electrode. Cyclic voltammetry (CV) was used to assess the working electrode surface. The electrode was placed in 1.5 mM ferrocene monocarboxylate (Fc) and the resulting oxidation plateau current was compared to the theoretical value (8.29 nA).

#### Biosensor Fabrication

Following fabrication of the combined needle microelectrodes, they were functionalized into biosensors as described elsewhere.^22,33^ Briefly, the working electrode was first coated with a poly(*m*-phenylenediamine) (mPD) layer, an exclusion layer to block out potential interferents that may otherwise be oxidized. Electropolymerization was used to coat the mPD onto the working electrode surface by placing the electrode in a 100 mM solution of mPD monomer in 0.01 M PBS and applying a potential of +0.7 V vs Ag|AgCl for 20 min. After electropolymerization, the electrode was left in solution for 5 min to allow stabilization of the mPD film before being removed and carefully rinsed with deionized water. A CV in 1.5 mM Fc was performed and the coating deemed successful if no oxidation peak could be seen. Following successful polymerization of the mPD film, the electrode was coated with a hydrogel containing the relevant enzyme.^22,23^ Different hydrogel compositions were used depending on the type of biosensor (glucose, lactate or glutamate), adapted from Vasylieva et al.^34^ as shown in Table 2.

**Table 2:** Composition of the GOx, LOx, and GlutOx Hydrogel Solutions

	glucose oxidase (GOx) hydrogel	lactate oxidase (LOx) hydrogel	glutamate oxidase (glutOx) hydrogel
	concentration (mg/mL)		
enzyme (GOx, LOx or GlutOx)	60	60	50
BSA	30	30	81
PEG-DE	14.8	45.6	78.0
glycerol	0.0125	0.0250	0.0125

The electrode was dipped in the relevant hydrogel for 90 s, before being placed in the oven for 2 h at 55 °C to allow cross-linking of the hydrogel matrix and immobilization of the enzyme. A diffusion-limiting polyurethane coating was added to the biosensor surface by dip-coating to extend the dynamic range of the biosensor. Following fabrication the biosensors were stored at −20 °C.

The biosensors work by the enzyme oxidizing the specific substrate to produce hydrogen peroxide, which is oxidized at the working electrode by applying a +0.7 V vs Ag|AgCl reference electrode and the resulting current detected.

#### Complementary Metal-Oxide Semiconductor (CMOS) Chip

In this work, the ion monitoring system was based on CMOS technology, and more specifically on an array of ion sensitive field effect transistors (ISFETs). The 78 × 56 ISFET array is part of a 4 mm × 4 mm chip fabricated by the Austrian Micro Systems foundry (AMS) in 0.35 μm CMOS technology, with a passivation layer made of 2 μm SiO_2_ coated with 2 nm of high-κ dielectric material by atomic layer deposition (ALD). The pixel architecture has been described elsewhere,^35^ and integrates the analogue sensing ISFET with a digital memory to enable in-pixel quantization and compensation of pixel offset due to trapped charges. The CMOS chips were encapsulated on 3.12 cm × 4.84 cm printed circuit board (PCB) cartridges.

#### ISM Functionalization

The surface of the CMOS chip was partially coated with a potassium ion selective membrane (ISM). The valinomycin-based potassium ISM was adapted from a standard protocol.^36^ The ISM was based on a poly(vinyl chloride) polymeric matrix, containing 0.92% (w/w) potassium ionophore I, 0.09% (w/w) potassium tetrakis(4-chlorophenyl) borate and 68.74% (w/w) bis(2-ethylhexyl) sebacate. The surface functionalization protocol has been described elsewhere.^24^ In summary, the ISM was diluted in 1 mL tetrahydrofuran and manually drop-cast onto the surface of the array in small droplets (typically 0.5 μL). After deposition, the membranes were left to dry for 30 min.

#### Microfluidic Flow Cells

For online measurements the biosensors were placed into a 3D printed biosensor flow cell, described elsewhere.^22^ The biosensor flow cell used in this study has a 350 μm × 350 μm channel running through the center. To insert the biosensors into the flow cell, they were placed inside 3D printed holders prior to functionalization and secured in place using M3 grub screws such that the biosensor tip would be positioned in the middle of the microfluidic channel when the holder was screwed into the biosensor flow cell.

The ion monitoring system used a separate microfluidic flow cell, described in more detail in Figure SI4. This flow cell design, referred to as the CMOS flow cell, has a circular channel of 350 μm in diameter and a chamber that sits on top of the CMOS chip with a volume of 0.5 μL. The total internal volume of this flow cell is 2.9 μL, which guarantees a good response time of the CMOS chip as well as any sensor connected downstream, as showed in Figure SI5. The CMOS flow cell integrates a custom-made Ag|AgCl reference electrode. After assembling the microfluidic CMOS flow cell over the chip, the ISM was conditioned overnight with artificial cerebrospinal fluid solution (aCSF).

#### Combined System

Both the CMOS flow cell and biosensor flow cell incorporated LabSmith CapTite bonded-port connectors to allow the flow cells to be connected together and to allow connection to the MD probe. Experiments were conducted with a one MD probe configuration or occasionally with a two MD probe configuration. In the one MD probe configuration the microfluidic flow cells were arranged such that the CMOS flow cell was connected to the outlet of the MD probe and the biosensor flow cell was connected to the outlet of the CMOS flow cell, using polyetheretherketone (PEEK) tubing (CAP360–150P, LabSmith, US), as shown in Figure 1. On occasions, connecting the two low-volume flow cells together led to back pressure issues, in which case a two MD probe configuration was used to prioritize the glucose measurement. In this setup, the CMOS flow cell was connected to the outlet of one MD probe and the biosensor flow cell was connected to the outlet of the other MD probe, both with PEEK tubing (CAP360–150P, LabSmith, US). In both configurations, the length of PEEK tubing between the MD probe and flow cell was approximately 0.5 m.

#### Wireless Monitoring System

Wireless potentiostats, described elsewhere,^21,23^ were used to apply a +0.7 V potential vs Ag|AgCl to the working electrode of the biosensors. Briefly, the wireless potentiostats were implemented on a 2-layer battery-powered (3.7 V rechargeable lithium-ion battery, 1.8 Ah, BAK) PCB, designed in-house. Each potentiostat has 2 amperometric channels, with a combination of 3 different gain options (1, 0.1 and 0.2 or 0.4 nA/V). The current was sampled at 10 Hz and sent in real time via Bluetooth to a Samsung tablet (SAMSUNG Galaxy Tab A 9.7″) and displayed using a custom-built Android app. To monitor the three analytes, glucose, lactate and glutamate, two potentiostats were required, which were held in a customized 3D printed case.

The PCB cartridges encapsulating the CMOS chips were integrated into a wireless and battery-powered (3.7 V lithium-ion rechargeable battery pack, 7.8 Ah, RS PRO) motherboard that communicated via Bluetooth to a Windows laptop with a customized app developed using Matlab App Designer for real-time visualization of the data, which is described in more details in the SI. The electronic board was embedded into a hand-held customized case.

#### Microfluidic Workstation

The microfluidic workstation was modified from the biosensor calibration platform described elsewhere.^37^ The workstation was adapted to allow the calibration of multiple analytes. Briefly, it consisted of a LabSmith breadboard, 20 μL syringe pumps, reservoirs and 2-way valves connected together with PEEK tubing (150 μm i.d., 360 μm o.d.). The syringe pumps were filled with different calibration standards, and the flow streams of each met at a mixing junction. The relative flow rate of each pump was varied to vary the concentration of each analyte at the analysis system downstream, with the total flow rate maintained at 1 μL/min to match the dialysate flow rate. A 4-way LabSmith valve was used to allow continuous flow. This ensured a smooth calibration trace as otherwise flow artifacts would occur due to pumps refilling. It also reduced the risk of air bubbles occurring and getting stuck within the flow channels of the two microfluidic flow cells. Examples of calibration traces and calibration curves obtained during the calibration phase of the sensors, prior to the start of the cardiac intervention protocol, are shown in Figures SI6 and SI7.

#### Microdialysis Probes

For this study, MAB 7.80.10 animal MD probes (Microbiotech, Sweden) were used for the online MD, with a molecular weight cutoff of 15 kDa and membrane length of 10 mm. Prior to insertion, the MD catheters were perfused with aCSF using a CMA107 pump (MDialysis, Sweden) at a flow rate of 1 μL/min. MD probes were implanted on average 3 h before baseline measurement commenced. After use, the MD probe tubing was wiped with ethanol and the membrane was rinsed with ethanol before being soaked in sterile perfusate fluid. Finally, the MD probe was perfused with sterile perfusion fluid. In most cases, a new MD probe was used each time, however, provided the probe had not been damaged during removal from the pig brain, the probes were sometimes reused.

CMA70 MD probes (MDialysis, Sweden) with a molecular weight cutoff of 20 kDa and membrane length of 10 mm were used for discontinuous offline MD. Prior to insertion, the MD catheters were perfused with aCSF using a CMA107 pump at a flow rate of 2 μL/min. Dialysate was collected in vials at set time intervals, determined by the cardiac protocol. After use, the MD probe was soaked in perfusate fluid. The probe was reused provided it was not damaged.

#### Animal Protocol

All procedures were approved by the Institutional Animal Care and Use Committee of the Medical University Innsbruck and the Austrian Ministry of Science, Research and Economy (protocol number BMWFW-2021–0.895.386). The study was conducted at the Experimental Research Unit of the Department of Anaesthiology and Intensive Care Medicine of the Medical University Innsbruck. Experiments were conducted in compliance with EU regulations for animal experimentation (Directive 2010/63/EU of the European Parliament and the Euoropean Council). Male and female pigs weighing in the range of 46–63 kg were anesthetized, intubated and ventilated as described elsewhere.^18^ A burr hole procedure was performed to allow the insertion of multiple probes including the offline and online MD probes and the brain tissue oxygen catheter (LICOX, Sanova Pharma GmbH, Vienna, Austria) into the cortex of the brain. The MD probes were inserted under direct vision into the same hemisphere; the online probe was inserted via a frontal burr hole and the offline probe was inserted via a parietal burr hole.

#### Cardiac Arrest and CPR

The experimental protocol is outlined in Figure 1. Baseline measurements were carried out for a minimum of 30 min. Cardiac arrest was induced by applying a 50 Hz, 60 V alternating current via two electrodes. This was followed by a period of 5 min where no intervention took place. Following this, CPR was started including rescue ventilations and mechanical chest compressions, initiating a 3 min cycle. On minute one adrenaline was administered, on minute two the heart was defibrillated, and on minute three mechanical chest compressions were briefly paused to check for ROSC. This cycle would repeat for up to 15 min until ROSC was achieved. If ROSC was not achieved, the pigs were euthanized by intravenous administration of potassium chloride. If ROSC was achieved, monitoring continued for 2 h before the pigs were euthanized.

## Supporting Information Available

Brain tissue oxygenation in one case through the experimental protocol, Raw blood glucose measurements for one case through the experimental protocol, Blood potassium measurements for one case through the experimental protocol, Additional details of CMOS microfluidic flow cell, Information on temporal response of overall system, Example calibration data for each of the sensors, Information on the ion monitoring app (PDF)

## Notes

For the purpose of open access, the authors have applied a Creative Commons Attribution (CC BY) license.
