# Supplementary material for: The Neurochemical Signature of Cardiac Arrest: A Multianalyte Online Microdialysis Study
Source: ACS Chem Neurosci. 2025 Mar 18;16(7):1323–34. doi: 10.1021/acschemneuro.4c00777 (PMC11969431; doi:10.1021/acschemneuro.4c00777)
Supplement: Supplementary file 1 — cn4c00777_si_001.pdf [file cn4c00777_si_001.pdf]

SUPPORTING INFORMATION

The neurochemical signature of cardiac arrest: a multi-analyte online microdialysis study

Cicatiello, C.,[1‡] Gowers, S. A. N.,[1‡] Smith, G. K.,[1] Pinggera, D.,[2] Orlob, S.,[3,4] Wallner, B.,[5] Schiefecker, A.,[6] Moser, N.,[7] Georgiou, P.,[7] Helbok, R.,[6,8,9] Martini, J.,[5] Putzer, G.,[5,10] Boutelle, M. G.[1*]

1. Department of Bioengineering, Imperial College London, London, SW7 2AZ, UK
2. Department of Neurosurgery, Medical University of Innsbruck, Innsbruck, 6020, Austria
3. Department of Anaesthesiology and Intensive Care Medicine, Medical University Graz, Graz, 8010, Austria
4. Institute for Emergency Medicine, University Hospital Schleswig-Holstein, Kiel, 24105, Germany
5. Department of Anaesthesia and Intensive Care Medicine, Medical University of Innsbruck, Innsbruck, 6020, Austria
6. Department of Neurology, Medical University of Innsbruck, Innsbruck, 6020, Austria
7. Department of Electrical and Electronic Engineering and Institute of Biomedical Engineering, Imperial College London, London, SW7 2AZ, UK
8. Department of Neurology, Kepler University Hospital, Johannes Kepler University Linz, Linz, 4020, Austria
9. Clinical Research Institute of Neuroscience, Johannes Kepler University Linz, Kepler University Hospital, Linz, 4020, Austria
10. Department of Cardiac Anaesthesiology and Intensive Care Medicine, Deutsches Herzzentrum der Charité (DHZC), Berlin, 10117, Germany

Corresponding author: m.boutelle@imperial.ac.uk

Brain tissue oxygenation

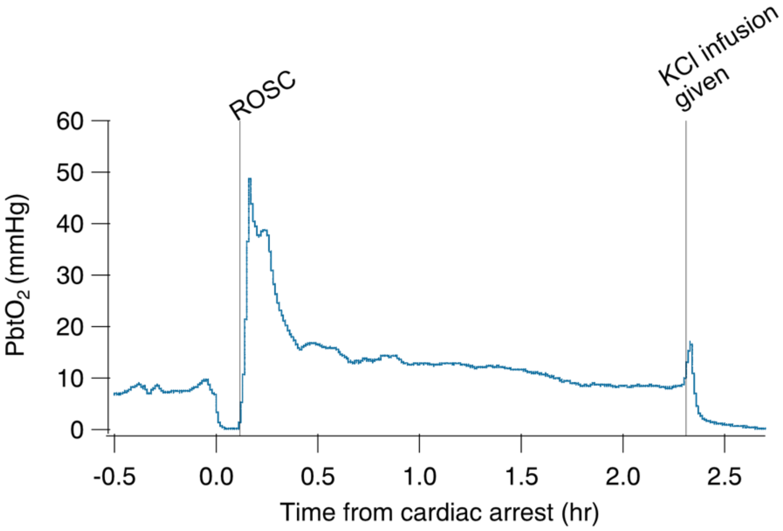

Figure SI1. Typical trend of brain tissue oxygen tension (PbtO2) during the cardiac intervention protocol. The brain tissue oxygenation was continuously monitored with a brain tissue oxygen catheter (LICOX, Sanova Pharma GmbH, Vienna), implanted in the same burr hole as the other MD probes. The vertical lines indicate the time of ROSC and the time of the infusion of potassium chloride (KCl).

Blood measurements

Figure SI1 shows the arterial and venous glucose measurements taken during the cardiac intervention protocol in comparison with the continuous monitoring of glucose levels in the brain dialysate.

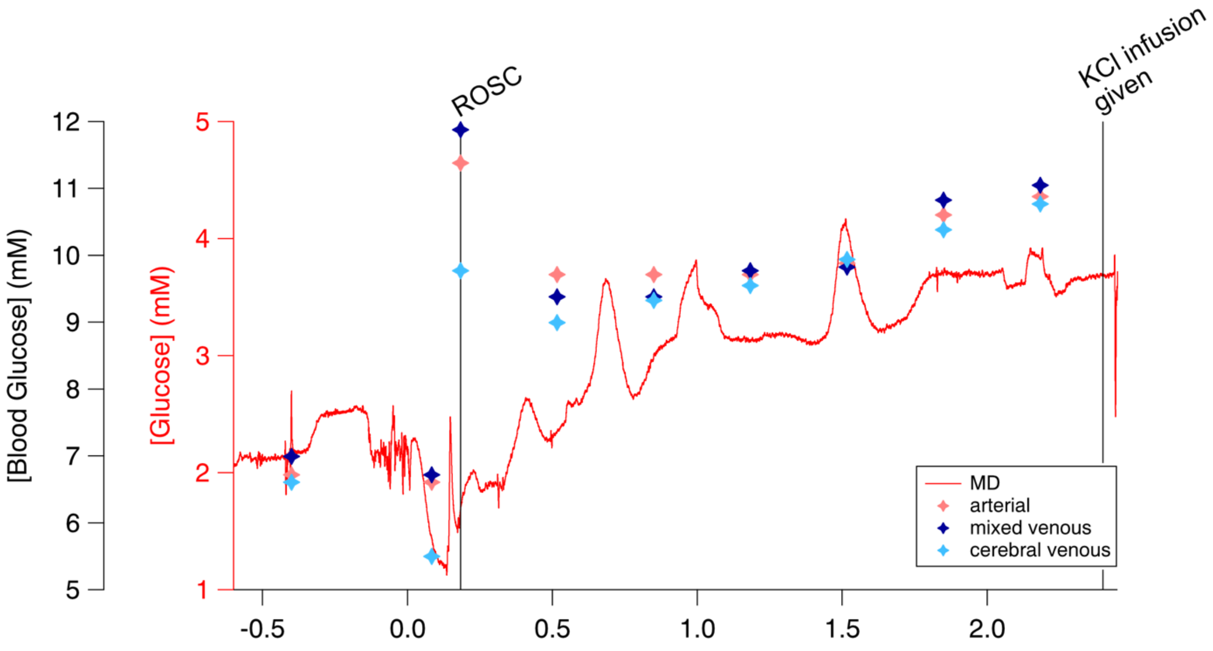

Figure SI2. Comparison of glucose levels in the dialysate with that of arterial and venous blood samples. The continuous MD data for glucose (in red) is compared with the glucose levels in the arterial (in pink), mixed-venous (in blue) and cerebral-venous (in light blue) blood samples that were taken at regular intervals during the clinical procedure and measured using a blood gas analyser: at baseline, at the start of CPR, at ROSC, and afterwards every 20 minutes until the end of the procedure.

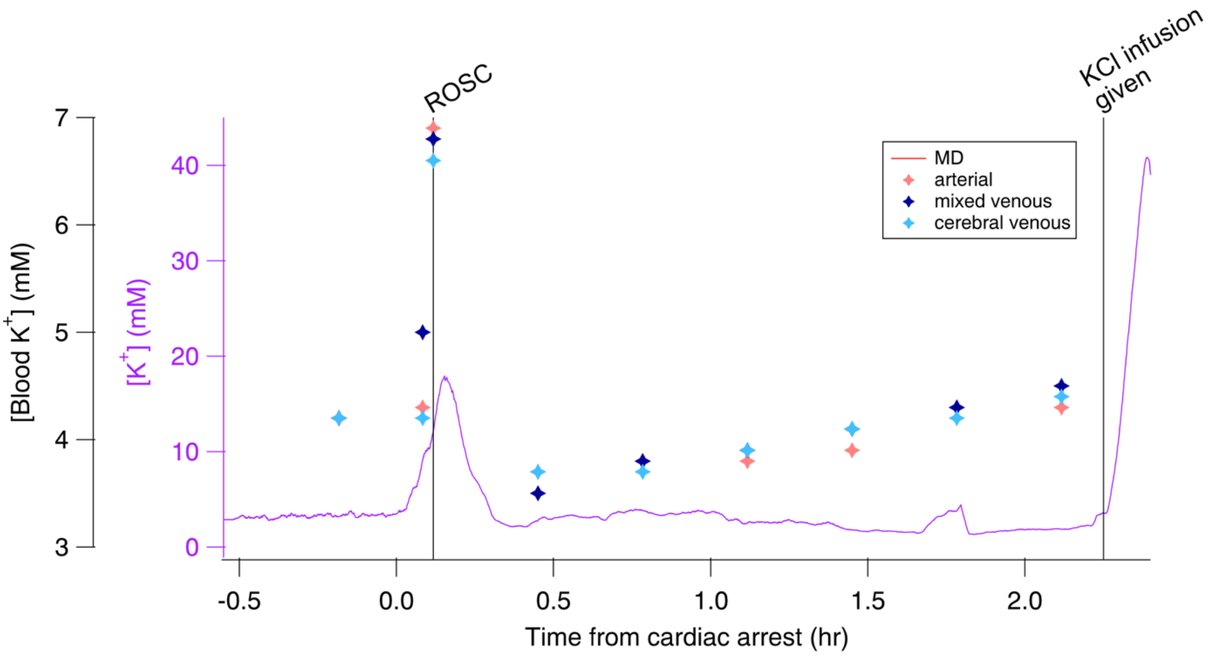

Figure SI3. Comparison of potassium levels in the dialysate with that of arterial and venous blood samples. The continuous MD data for potassium (in purple) is compared with the glucose levels in the arterial (in pink), mixed-venous (in blue) and cerebral-venous (in light blue) blood samples that were taken at regular intervals during the clinical procedure and measured using a blood gas analyser: at baseline, at the start of CPR, at ROSC, and afterwards every 20 minutes until the end of the procedure.

CMOS microfluidic flow cell

Figure SI4 shows the design and the assembly schematic for the CMOS microfluidic flow cell. The flow cell has a total internal volume of 2.9 µl, and has a circular channel of 350 µm in diameter and a chamber with a volume of 0.5 µl. The flow cell chamber sits on top of the CMOS chip, allowing the MD stream to be in contact with the ISM. This flow cell integrates a custom-made Ag|AgCl reference electrode fabricated using Ag|AgCl paste (SunChemical, USA) on 3D printed parts. The reference electrode was screwed into the flow cell channel through a compressible silicon gasket, which ensures a tight and leak-free connection. The microfluidic flow cells and the reference electrode parts were 3D printed with the ProJet MJP 2500/2500 Plus (3D System, USA) in VisiJet M2S-HT90 and in VisiJet M2S-HT250 materials, respectively. The flow cell was assembled onto the CMOS chip using an intermediate layer of a laser-cut medical grade double-sided tape (ARcare 90445), and a pair of M2 bolts, which were fastened through the side holes of the flow cell and PCB cartridge and secured with nuts on the opposite side. To facilitate a leak-free connection with the other microfluidic components, LabSmith CapTite bonded-port connectors were used at the inlet and outlet of the flow cell and glued in place using Araldite RAPID (Huntsman Advanced Materials LLC, USA).

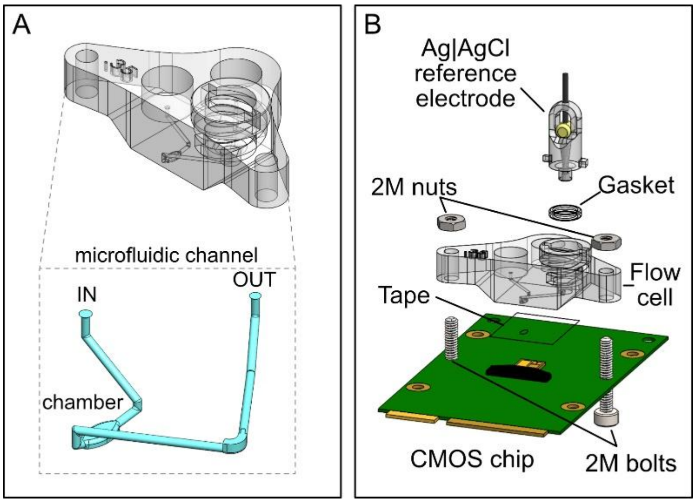

Figure SI4. CMOS microfluidic flow cell (A) 3D design of the microfluidic flow cell for the CMOS chip. (B) Schematic of the assembly: the flow cell was first attached to the CMOS chip using a piece of laser cut double sided tape so that the chamber was over the ISM and then secured using a pair of M2 bolts and nuts on the edges. The Ag|AgCl reference electrode was screwed into the microfluidic flow cell using a silicon gasket.

Time response for the combined system

The microfluidic design of the CMOS flow cell was optimised to minimise dead volume and to improve the response time of the CMOS chip as well as any sensors connected downstream. Figure SI5 shows the normalised response to a step change in concentration for the combined system consisting of the CMOS flow cell outlet connected to the inlet of the biosensor flow cell containing a glucose biosensor. The normalised response of the glucose biosensor is compared with that obtained using a different setup, where the biosensor flow cell was connected directly to the microfluidic workstation with a LabSmith CapTite interconnect union (without the CMOS flow cell first, labelled 'control') to investigate the effect of having the CMOS flow cell first on the response of the glucose biosensor. The T90 (the time required to reach 90% of a step change starting from 0%) for the glucose biosensor in the control setup was 2.05 ± 0.17 min (n = 6). In the combined system, the resulting T90 for the glucose biosensor was 2.36 ± 0.09 min (n = 8), while for the CMOS chip it was 1.09 ± 0.14 min (n = 5).

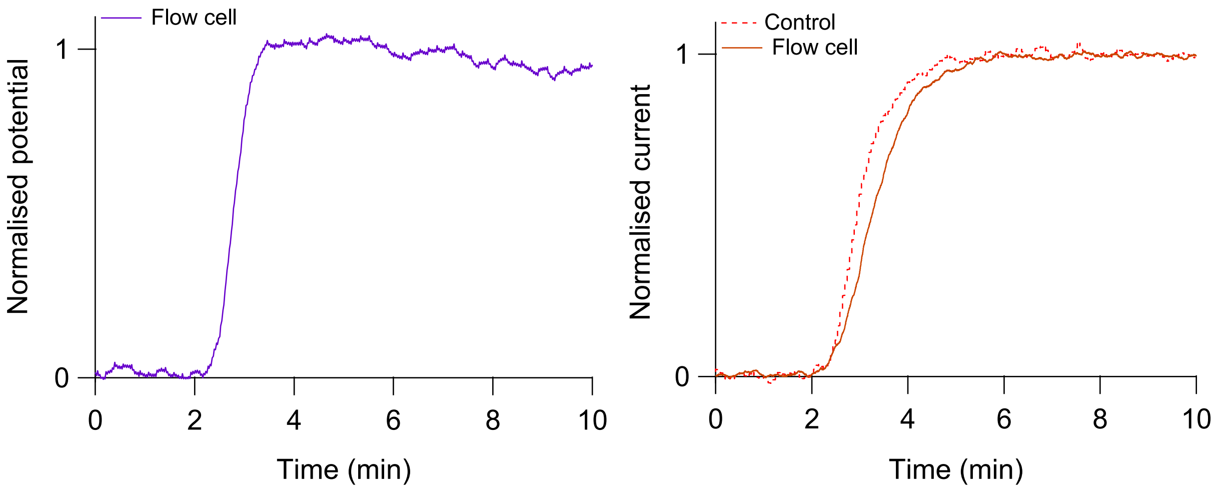

Figure SI5. Normalised response of the combined system. The two graphs show examples of the normalised responses of a CMOS chip coated with a potassium ISM (left) and a glucose biosensor (right). These responses are to a concentration step change from 2.7 mM to 27 mM for potassium and from 0 mM to 2 mM for glucose, at a flow rate of 1µl/min. In this setup the CMOS flow cell is connected directly to the microfluidic workstation and the biosensor flow cell containing a glucose biosensor is connected to the outlet of the CMOS flow cell. The control for glucose is obtained by connecting the biosensor flow cell directly to the microfluidic workstation with a LabSmith CapTite interconnect union.

Calibration curves

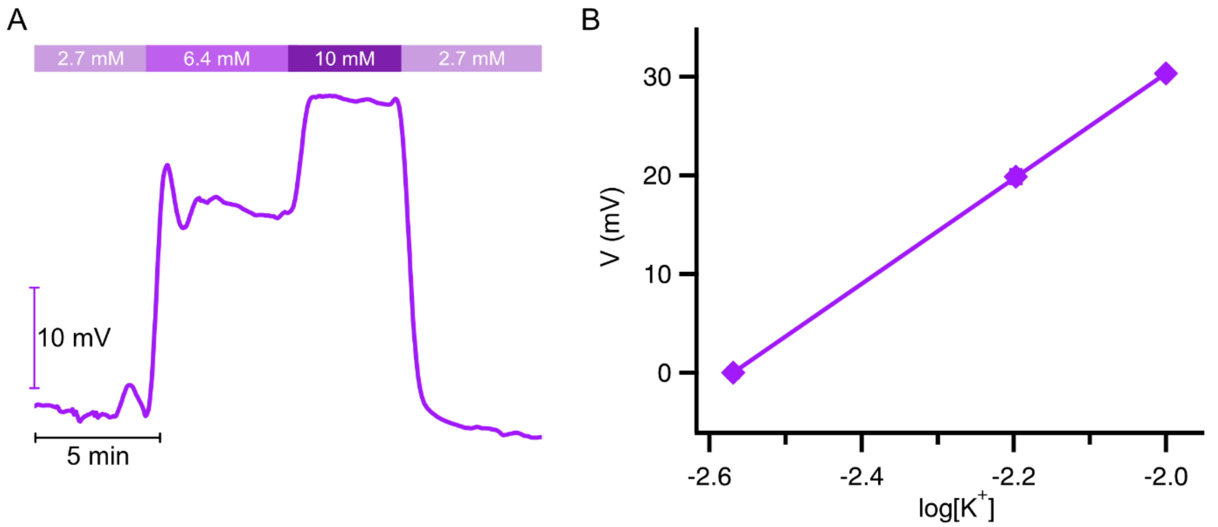

Figure SI6. Calibration of the potassium sensor before the start of the cardiac intervention protocol (A) Calibration trace during a 3-step calibration cycle at a flow rate of 1 µl/min in which potassium concentration was changed from 2.7 mM to 10 mM. The data were sampled at 0.3 Hz and were filtered using a Savitzky–Golay filter (25-point). (B) Normalised sensitivity curve. The markers and error bars represent the mean and the standard deviation of the normalised signal relative to the value at 2.7 mM potassium concentration, measured in intervals of 3 minutes (n = 80 data points). Weighted linear regression was used to fit the curves with a 95% confidence interval. The resulting sensitivity is of 53.34 ± 0.92 mV/dec.

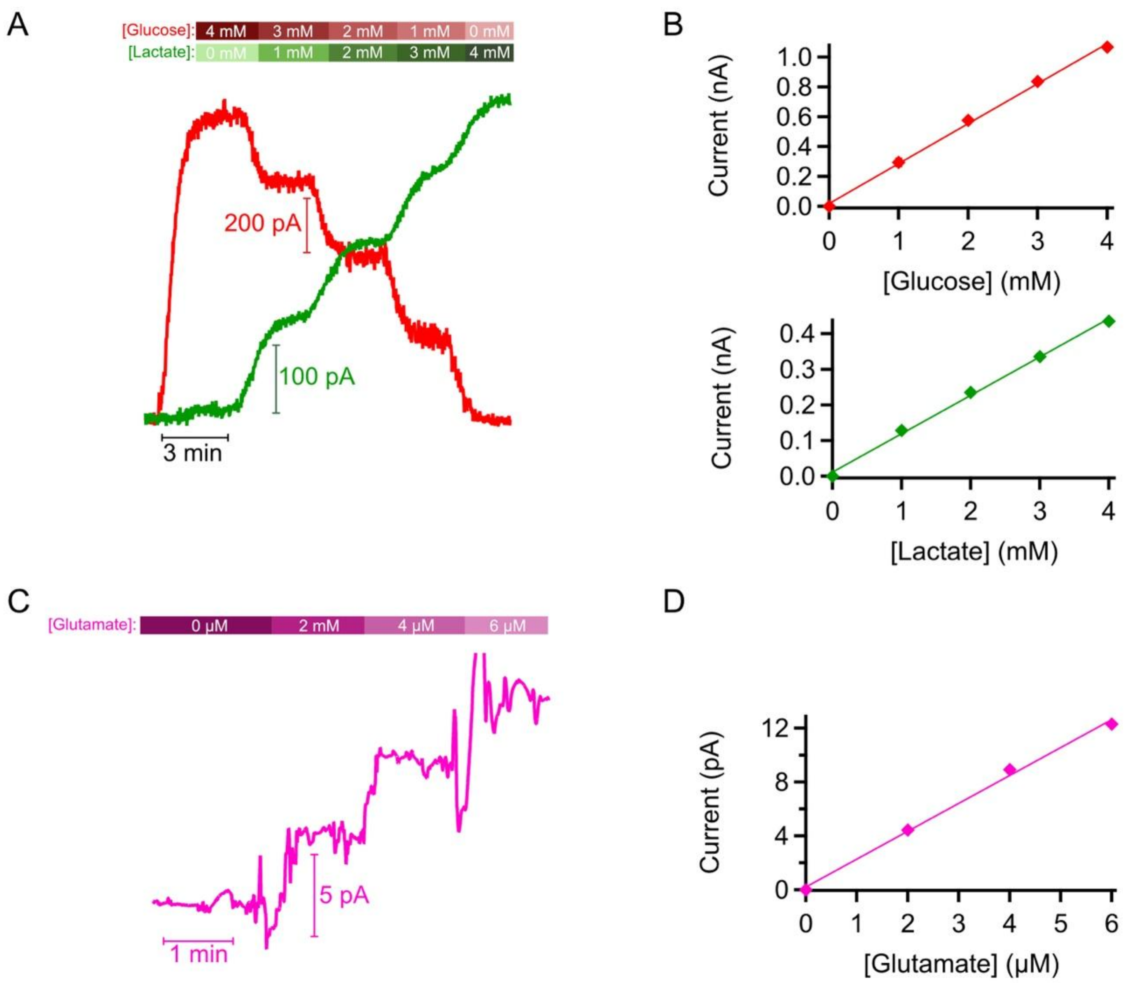

Figure SI7. Example calibrations of glucose, lactate and glutamate biosensors (A) Example 5-point calibration of glucose (red) and lactate (green) from 0 – 4 mM in 1 mM steps. The data were sampled at 10 points per second and smoothed with a 201-point Savitsky-Golay filter. (B) Corresponding calibration curves for glucose (red) and lactate (green) fitted with a straight line. Markers and error bars represent the mean and standard deviation of the signal at each step. For these sensors the glucose sensitivity was 0.27 ± 0.007 nA/mM and the lactate sensitivity was 0.11 ± 0.003 nA/mM (C) Example 4-point calibration of glutamate (pink) from 0 – 6 µM in 2 µM steps. The data were sampled at 200 points per second and smoothed with a 501-point Savitsky-Golay filter. (D) Corresponding calibration curve for glutamate fitted with a straight line. Markers and error bars represent the mean and standard deviation of the signal at each step. For this glutamate sensor the sensitivity was 2.1 ± 0.09 pA/mM.

Ion monitoring app

The ion monitoring app allowed online computation of the pixels that were in contact with the dialysate stream inside the CMOS flow cell chamber. The app presented the data in binary array images as well as in 2D plots showing the average response. During the sensor calibration, the user could start clustering algorithms with user-defined threshold values to distinguish the pixels that were coated with the ISM, and hence sensitive to potassium ions. The online clustering allowed online visualisation of the voltage response of the potassium pixels in real time during the experiment. The app saved the clustering results and the raw experimental data from the CMOS chip in a matrix where the number of rows is equal to the number of time frames recorded throughout the experiment, and the number of columns is equal to the number of pixels. This matrix was later used for further offline analysis.
